# Association between perfluoroalkyl and polyfluoroalkyl substances and adolescents' sleep disorders: NHANES 2005–2018

**DOI:** 10.3389/fnut.2025.1584281

**Published:** 2025-05-09

**Authors:** Bocheng Gao, Yanju Gong, Yan Lu, Shuhua Gou, Xingyue Lai, Gan Luo, Hong Yang

**Affiliations:** ^1^School of Medical and Life Sciences, Chengdu University of Traditional Chinese Medicine, Chengdu, China; ^2^School of Basic Medical Sciences, Chengdu University of Traditional Chinese Medicine, Chengdu, China; ^3^Department of Orthopedics, Chengdu Integrated Traditional Chinese Medicine & Western Medicine Hospital/Chengdu First People's Hospital, Chengdu, China

**Keywords:** NHANES, PFAS, PFOS, adolescents, sleep disorders

## Abstract

**Background:**

Previous research indicates that per- and polyfluoroalkyl substances (PFAS) can disrupt metabolism and neurological function via endocrine pathway interference and neuroinflammation. These effects may impair melatonin secretion and disrupt circadian rhythm regulation, suggesting potential links to sleep health. However, the impact of PFAS exposure on adolescent sleep remains unclear. This study examines the associations between PFAS exposure and sleep health indicators in U.S. adolescents.

**Methods:**

Data from 838 adolescents who participated in the 2005–2018 National Health and Nutrition Examination Survey (NHANES) were analyzed to investigate the association between PFAS exposure and physician-diagnosed sleep disorders. Eight PFAS compounds were identified. Multivariate logistic regression models, restricted cubic spline (RCS) curves, Bayesian kernel machine regression (BKMR), and weighted quantile sum (WQS) regression were used to assess single, linear, and combined effects on adolescent sleep disorders.

**Results:**

Negative associations were observed between adolescent sleep disorders and three PFAS compounds, specifically perfluorooctanoic acid (PFOA), perfluorooctanesulfonic acid (PFOS), and perfluorononanoic acid (PFNA). RCS analysis revealed a significant linear relationship (*P* for non-linear > 0.05). The BKMR and WQS models demonstrated a combined effect of PFAS exposure on sleep disorders, with PFOS demonstrating the most substantial contribution (effect size: 0.91). The stratified analysis revealed that PFOS exposure had a greater impact on females [odds ratio (OR): 0.54, 95% confidence interval (CI): 0.33–0.87] than males (OR: 0.50, 95% CI: 0.24–1.01), suggesting sex-specific differences in vulnerability.

**Conclusions:**

Our findings indicate a negative correlation between specific PFAS and specific sleep disorders in adolescents, with PFOS being the dominant effect component in the PFAS mixture and stronger effects observed in females. However, due to the cross-sectional nature of the study, a causal relationship cannot be established. These results highlight the potential public health impact of PFAS exposure and the need to further investigate the underlying mechanisms and causal pathways in future longitudinal or experimental studies.

## 1 Introduction

Perfluoroalkyl and polyfluoroalkyl substances (PFAS) are a class of highly fluorinated aliphatic compounds broadly used in consumer products including disposable food packaging, cookware, outdoor equipment, furniture, and carpets (1). Their hydrophobic and oleophobic properties render them environmentally persistent and difficult to avoid. These findings underscore the potential public health accumulation of PFAS in blood is a global phenomenon. For example, PFAS have been detected in 98% of Americans (2). In the European Union, adults have a median serum PFOS level of 5.3 μg/L, while in China, residents near e-waste recycling zones exhibit levels ranging from 4.8 to 12.7 μg/L (3). Exposure primarily occurs through dietary sources, drinking water, and environmental contact (4–6). PFAS exposure may have a complex association with the onset and progression of several neurodegenerative diseases. Notably, in Alzheimer's disease (AD), Parkinson's disease (PD), and multiple sclerosis (MS), PFAS may exacerbate the pathological processes through various mechanisms, including interference with the normal physiological functions of the nervous system, induction of neuroinflammatory responses, and disruption of neurotransmitter metabolism (7–9). Additionally, sleep disorders, which are highly prevalent in patients with neurodegenerative diseases, are closely related to disease progression and prognosis (10). Nevertheless, associations between PFAS exposure and sleep disorders have received limited attention in the existing literature.

Sleep disorders encompass a range of issues, including poor sleep quality and various sleep complaints (11, 12), and have become a significant public health concern among adolescents worldwide (13, 14). A meta-analysis estimated that 7%−36% of adolescent's worldwide experience sleep disorders (15). There are currently inconsistent conclusions in studies on the relationship between PFAS and sleep disorders. Li et al. (16) found that cumulative PFAS exposure could plausibly have adverse effects on sleep well. Another study identified a negative association between specific PFAS and sleep disorders in U.S. adults (12). In addition, adolescence is a critical developmental stage characterized by metabolic activity, physiological immaturity, and increased sensitivity to external factors (17), rendering this age group particularly vulnerable to potential PFAS-related health effects. However, there is a lack of research studying the relationship between PFAS and sleep disorders in adolescents.

Bayesian kernel machine regression (BKMR) uses kernel functions and Bayesian estimation to flexibly model non-linear relationships and interactions between mixture components and health outcomes without predefining parameter forms. Weighted quantile sum regression (WQS) integrates multiple factors' effects into a single index, reducing data dimensionality and multicollinearity. These methods overcome single-effect analysis limitations, comprehensively assess multiple factors' joint effects, enhance model stability and accuracy, and accurately reflect factor-outcome relationships. Therefore, we aimed to study the relationship between PFAS exposure and sleep disorder occurrence in adolescents and contribute to our understanding of potential environmental influences on adolescent sleep health.

## 2 Methods

### 2.1 Study design and demographics

The National Health and Nutrition Examination Survey (NHANES) is a nationally representative program conducted by the CDC in the United States. This program introduces advanced multistage sampling techniques to collect data through household interviews, physical examinations, and laboratory analyses. The survey gathers information on demographics, health-related factors, and physiological and biochemical parameters. NHANES data are released biennially and widely utilized in public health research and policy development.

**Figure 1:**
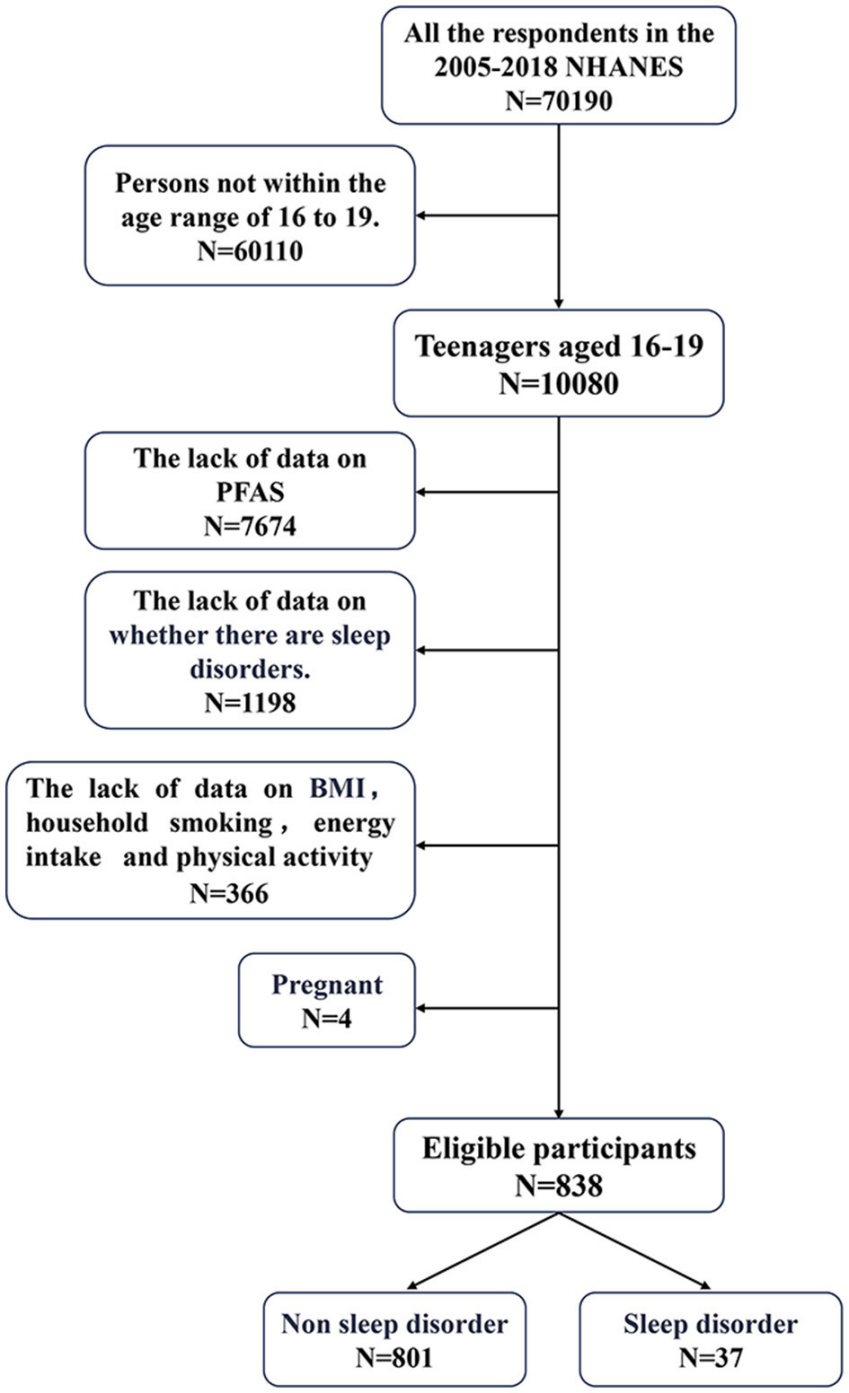
Exclusion criteria for the study population. PFAS, perfluoroalkyl and polyfluoroalkyl substances; NHANES, National Health and Nutrition Examination Survey; BMI, body mass index.

Data from seven NHANES cycles conducted between 2005 and 2018 were used for analyses. Of the 70,190 participants, 10,090 adolescents aged 12–19 years were identified. Of these, 7,674 were excluded owing to the absence of biological samples required for PFAS analysis. An additional 1,198 adolescents were excluded either because they did not report a sleep disorder or they declined to participate in the survey. Moreover, 366 participants were further excluded with missing covariate information and four pregnant adolescents to ensure data integrity. After applying these exclusion criteria, 838 adolescents were included in the final analysis. The detailed participant selection process is presented in Figure 1.

### 2.2 Assessment of PFAS serum concentration exposure

Blood samples taken from the participants were handled and kept in sample vials at −20°C. Subsequently, these samples were transferred to a laboratory designated by the CDC for analysis. The limit of detection (LOD) for each PFAS compound was established based on three times the standard deviation of the blank sample concentration (18). The detectable machine-generated result was recorded for values below the LOD. The LOD divided by the square root of 2 was substituted when no machine-readable values were available, following the NHANES guidelines.

### 2.3 Sleep disorders in adolescents

Data on sleep disorders were derived from responses to the NHANES questionnaire administered between 2005 and 2018. Participants were asked, “Has a doctor or another health professional ever told you that you have a sleep disorder?” Responses were used to categorize participants into two groups: those who answered “yes” were classified as having a sleep disorder, whereas those who answered “no” were classified as not having a sleep disorder.

### 2.4 Covariate evaluation

Covariates were chosen based on existing evidence and previous studies to account for potential confounders that might affect the relationship between PFAS exposure and adolescent sleep disorders. Data on personal demographics and lifestyle factors were obtained from the questionnaire, including age (in years), sex (male or female), and ethnic/racial background (Mexican American, Other Hispanic, Non-Hispanic Black, Non-Hispanic White, or Other Race, including Multi-Racial). Additionally, body mass index (BMI, kg/m2), the ratio of family income to poverty (PIR, <1.35, 1.35–3.00, ≥3.00), household smoking (yes or no), energy intake levels (adequate, high, or low), and physical activity (PA) levels (active or inactive) were recorded. Energy intake represents the mean of individual 24 h and 48 h dietary recalls. The amount of PA (MET h/week) was calculated based on leisure-time PA types, leisure-time PA times, and leisure-time PA frequency in a week. These variables were included to provide a comprehensive description of the study population and facilitate subgroup analysis.

### 2.5 Statistical analyses

#### 2.5.1 Analysis of individual PFAS

Demographic characteristics of the participants were summarized using descriptive statistics. Adolescents were categorized into sleep disorders and non-sleep disorder groups. Continuous variables were reported as means with standard deviation (SD) for each group, whereas categorical variables were presented as frequencies with percentages. A *t*-test was used to measure the difference between groups, whereas the Wilcoxon rank-sum test was used for non-normally distributed variables. Categorical variables were analyzed using the chi-squared test.

Logarithmic transformations were performed to normalize the data for subsequent analyses owing to the non-normal distributions of PFAS concentrations. Pearson correlation coefficients were determined for the log-transformed PFAS concentrations to evaluate the inter-relationships among biomarkers. Logistic regression was employed to evaluate the potential association between PFAS levels and sleep disorders. Three models were developed for the analysis: Model 1 included no covariate adjustments; Model 2 was adjusted for age, sex, race, and BMI; and Model 3 was adjusted for covariates including sex, age, race, PIR, BMI, household smoking status, energy intake and PA. Restricted cubic spline (RCS) curves were generated to examine the potential relationship between PFAS levels and sleep disorders for three specific PFAS compounds: perfluorooctanoic acid (PFOA), perfluorooctanesulfonic acid (PFOS), and perfluorononanoic acid (PFNA). Subgroup analyses were conducted by stratifying participants according to covariates to evaluate associations across different adolescent subpopulations.

#### 2.5.2 Combined effects

To evaluate the combined effects of PFAS mixtures on sleep disorders, we applied two distinct methodologies to model and parameterize exposure: BKMR and WQS regression. BKMR was used to capture potential non-linear relationships and interactions among PFAS compounds, whereas WQS regression was used to quantify the combined impact of PFAS mixtures on sleep disorders.

All statistical analyses were performed using R version 4.4.0 (R Core Team), with mixed-method analyses performed using the “bkmr” and “gWQS” packages. A two-sided *P-value* < 0.05 was considered to be statistically significant.

## 3 Results

### 3.1 General demographics

The demographic characteristics and PFAS levels of the 838 adolescents comprising 450 males and 388 females are summarized in Table 1. Among them, 801 (95.58%) participants reported no sleep disorders, whereas 37 (4.42%) reported having a sleep disorder. No significant differences were observed between the sleep disorder and non-sleep disorder groups regarding race, socioeconomic status, BMI, household smoking status, energy intake, or PA levels (*P* > 0.05). However, significant variations in PFAS levels were identified. Adolescents without sleep disorders exhibited higher concentrations of specific PFAS compounds compared to those with sleep disorders. Significant differences were observed in PFOA (3.15 ng/mL vs. 2.19 ng/mL, *P* = 0.001), PFOS (10.6 ng/mL vs. 5.88 ng/mL, *P* < 0.001), and PFNA (1.05 ng/mL vs. 0.82 ng/mL, *P* = 0.025) levels between the non-sleep disorder and sleep disorder groups, respectively. No significant associations were observed between the two groups for perfluorohexane sulfonic acid (PFHxS), 2-N-methyl-perfluorooctane sulfonamidoacetic acid (Me-PFOSA-AcOH), perfluorodecanoic acid (PFDA), perfluoroundecanoate (PFUA), or perfluorododecanoate (PFDoA) (*P* > 0.05).

**Table 1:** Summary of characteristics of participants by sleep disorder status.

**Characteristic**	**Overall (*N* = 838)**	**Non-sleep disorder (*N* = 801)**	**Sleep disorder (*N* = 37)**	***P-value***
Sex				0.070
Male	450 (53.7%)	436 (54.4%)	14 (37.8%)	
Female	388 (46.3%)	365 (45.6%)	23 (62.2%)	
Age (years)	17.5 (1.12)	17.5 (1.13)	17.4 (1.07)	0.771
Race				0.591
Mexican American	233 (27.8%)	222 (27.7%)	11 (29.7%)	
Other Hispanic	73 (8.71%)	72 (8.99%)	1 (2.70%)	
Non-Hispanic White	243 (29.0%)	232 (29.0%)	11 (29.7%)	
Non-Hispanic Black	217 (25.9%)	208 (26.0%)	9 (24.3%)	
Other Race–including Multi-Racial	72 (8.59%)	67 (8.36%)	5 (13.5%)	
PIR				0.640
<1.35	368 (43.9%)	349 (43.6%)	19 (51.4%)	
1.35–3.00	252 (30.1%)	242 (30.2%)	10 (27.0%)	
≥3.00	218 (26.0%)	210 (26.2%)	8 (21.6%)	
BMI				0.111
Under 25	473 (56.4%)	458 (57.2%)	15 (40.5%)	
25–30.0	187 (22.3%)	177 (22.1%)	10 (27.0%)	
30.0 and over	178 (21.2%)	166 (20.7%)	12 (32.4%)	
Household smoking				1.000
Yes	150 (17.9%)	143 (17.9%)	7 (18.9%)	
No	688 (82.1%)	658 (82.1%)	30 (81.1%)	
Energy intake				0.894
Adequate	357 (42.6%)	340 (42.4%)	17 (45.9%)	
High	181 (21.6%)	173 (21.6%)	8 (21.6%)	
Low	300 (35.8%)	288 (36.0%)	12 (32.4%)	
Physical activity				0.494
Active	575 (68.6%)	552 (68.9%)	23 (62.2%)	
Inactive	263 (31.4%)	249 (31.1%)	14 (37.8%)	
PFOA	3.11 (2.01)	3.15 (2.02)	2.19 (1.57)	0.001
PFOS	10.4 (9.48)	10.6 (9.60)	5.88 (4.27)	<0.001
PFHxS	2.71 (3.59)	2.74 (3.62)	1.98 (2.78)	0.117
Me-PFOSA-AcOH	0.35 (0.41)	0.35 (0.41)	0.30 (0.39)	0.480
PFDA	0.29 (0.41)	0.29 (0.40)	0.33 (0.57)	0.677
PFNA	1.04 (0.70)	1.05 (0.70)	0.82 (0.59)	0.025
PFUA	0.17 (0.34)	0.17 (0.34)	0.19 (0.30)	0.763
PFDoA	0.11 (0.06)	0.11 (0.06)	0.11 (0.13)	0.824

**Figure 2:**
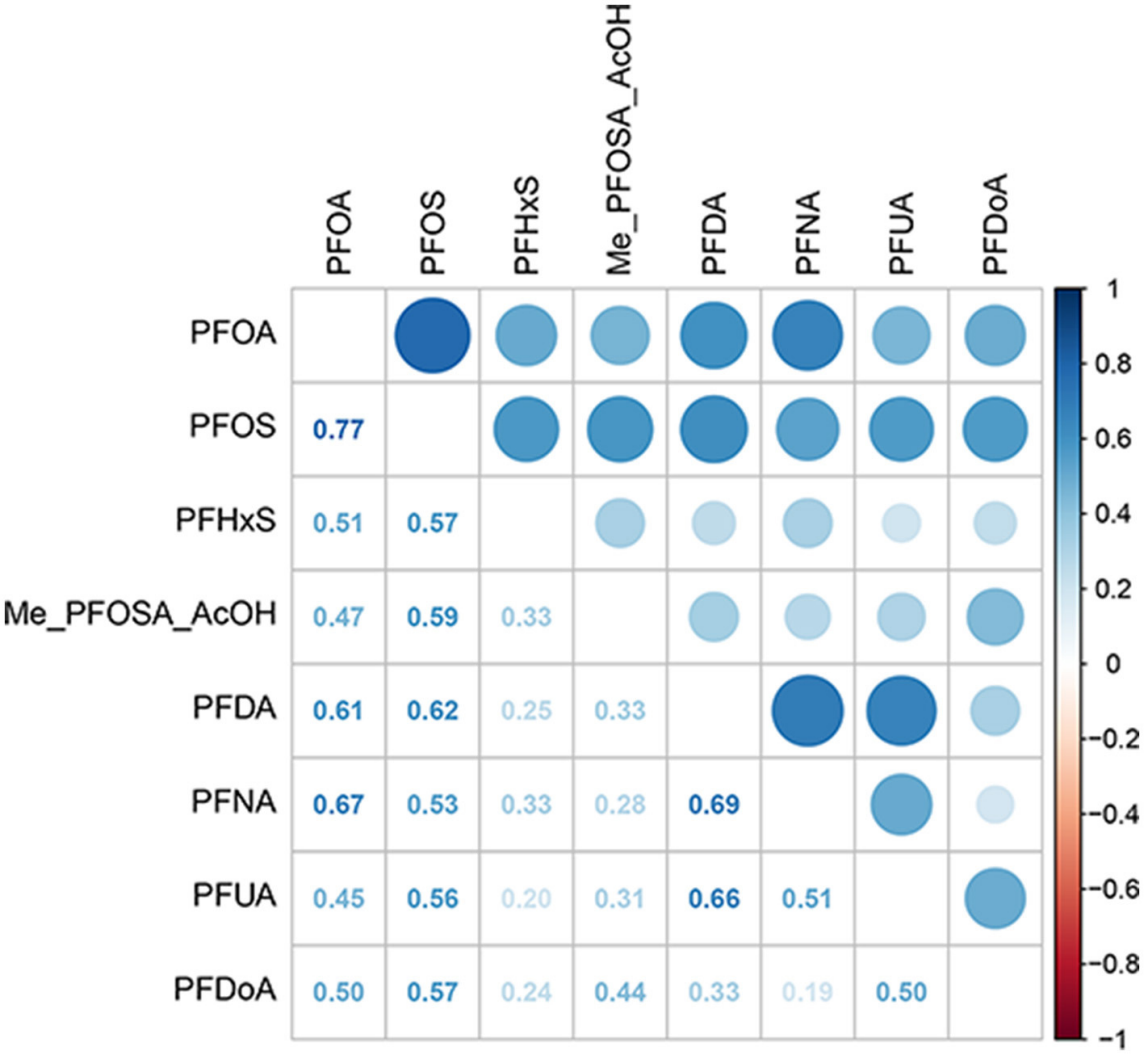
Heat map of PFAS correlations. PFOA, perfluorooctanoic acid; PFOS, perfluorooctane sulfonic acid; PFDA, perfluorodecanoic acid; PFHxS, perfluorohexane sulfonic acid; PFNA, perfluorononanoic acid; PFUA, perfluoroundecanoate; PFDoA, perfluorododecanoate; Me-PFOSA-AcOH, 2-N-methyl-perfluorooctane sulfonamidoacetic acid.

The distributions of serum concentrations for the eight analyzed PFAS compounds in this study are presented in Table 2. Among them, PFOS had the highest median concentration at 7.50 ng/mL, with PFOA following at 2.62 ng/mL. NO statistically significant correlations were observed between serum PFAS (Figure 2), indicating that the concentrations of these PFAS compounds were independent of each other.

**Table 2:** Distribution of PFAS concentrations.

**PFAS**	**Mean**	**SD**	**Min**	**P10**	**P25**	**P50**	**P75**	**P90**	**Max**
PFOA	3.11	2.01	0.07	1.09	1.65	2.62	4.10	5.50	15.20
PFOS	10.38	9.48	0.32	2.50	4.10	7.50	13.30	21.19	100.00
PFHxS	2.71	3.59	0.07	0.48	0.80	1.50	3.10	5.96	42.40
Me_PFOSA_AcOH	0.35	0.41	0.06	0.06	0.10	0.20	0.44	0.80	4.10
PFDA	0.29	0.41	0.07	0.07	0.14	0.20	0.30	0.50	8.50
PFNA	1.04	0.70	0.07	0.40	0.60	0.90	1.31	1.80	5.99
PFUA	0.17	0.34	0.07	0.07	0.07	0.14	0.14	0.30	8.80
PFDoA	0.11	0.06	0.07	0.07	0.07	0.07	0.14	0.14	0.90

### 3.2 Associations of individual PFAS compounds with sleep disorders

The association between PFAS and sleep disorders was valuated with logistic regression analysis (Table 3). All tested PFAS compounds demonstrated a significant negative correlation with sleep disorders in unadjusted Model 1, with PFOA, PFOS, PFHxS, and PFNA showing particularly strong significance (*P* < 0.05). Specifically, PFOA was significantly associated with a decreased risk of sleep disorders (OR: 0.51, 95% CI: 0.38–0.70, *P* < 0.001), inferring that higher exposure to PFOA may potentially play a protective role against sleep disorders. The negative associations for PFOA, PFOS, and PFNA remained statistically significant in Model 2 adjusted for age, sex, race, and BMI. In Model 3 further adjusted for socioeconomic and lifestyle factors, including PIR, household smoking status, energy intake, and physical activity, PFOA (OR: 0.53, 95% CI: 0.38–0.75, *P* < 0.001), PFOS (OR: 0.55, 95% CI: 0.38–0.79, *P* = 0.002), and PFNA (OR: 0.64, 95% CI: 0.46–0.89, *P* = 0.009) were also significantly associated with sleep disorders. In contrast, other PFAS compounds, including PFHxS, Me-PFOSA-AcOH, PFDA, PFUA, and PFDoA, displayed weak or non-significant associations across all models (*P* > 0.05).

**Table 3:** Association between PFAS and sleep disorders in the different logistic regression models.

**PFAS**	**^a^Model 1**	***P-value***	**^b^Model 2**	***P-value***	**^c^Model 3**	***P-value***
PFOA	0.51 (0.38, 0.70)	<0.001	0.52 (0.37, 0.72)	<0.001	0.53 (0.38, 0.75)	<0.001
PFOS	0.55 (0.40, 0.76)	<0.001	0.55 (0.39, 0.78)	<0.001	0.55 (0.38, 0.79)	0.002
PFHxS	0.70 (0.50, 0.97)	0.031	0.72 (0.50, 1.02)	0.067	0.71 (0.49, 1.03)	0.066
Me_PFOSA_AcOH	0.72 (0.50, 1.01)	0.067	0.71 (0.49, 1.02)	0.072	0.73 (0.49, 1.05)	0.097
PFDA	0.71 (0.49, 1.01)	0.062	0.71 (0.49, 1.01)	0.065	0.74 (0.50, 1.06)	0.109
PFNA	0.60 (0.44, 0.83)	0.002	0.63 (0.46, 0.88)	0.006	0.64 (0.46, 0.89)	0.009
PFUA	0.83 (0.56, 1.17)	0.333	0.82 (0.56, 1.16)	0.302	0.86 (0.57, 1.21)	0.413
PFDoA	0.72 (0.49, 1.03)	0.089	0.74 (0.49, 1.05)	0.111	0.72 (0.48, 1.05)	0.105

a Model 1 unadjusted.

b Model 2 adjusted for age, sex, race, and body mass index.

c Model 3 adjusted for the ratio of family income to poverty, household smoking, energy intake, and physical activity based on Model 2.

**Figure 3:**
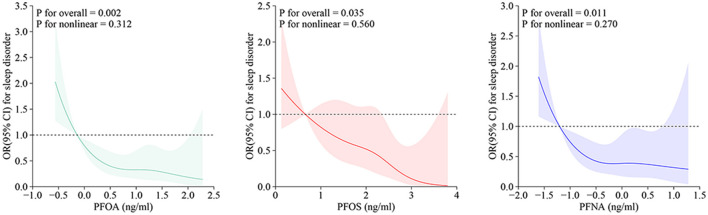
RCS curves for PFAS and adolescent sleep disorders. OR, odds ratio; CI, confidence interval; PFOA, perfluorooctanoic acid; PFOS, perfluorooctane sulfonic acid; PFNA, perfluorononanoic acid.

RCS analysis revealed a significant linear association between serum concentrations of PFOA, PFOS, and PFNA and sleep disorder risk among adolescents (*P* for non-linearity > 0.05), indicating that there was no threshold effect (Figure 3).

### 3.3 Association between PFAS mixtures and sleep disorders in adolescents

**Figure 4:**
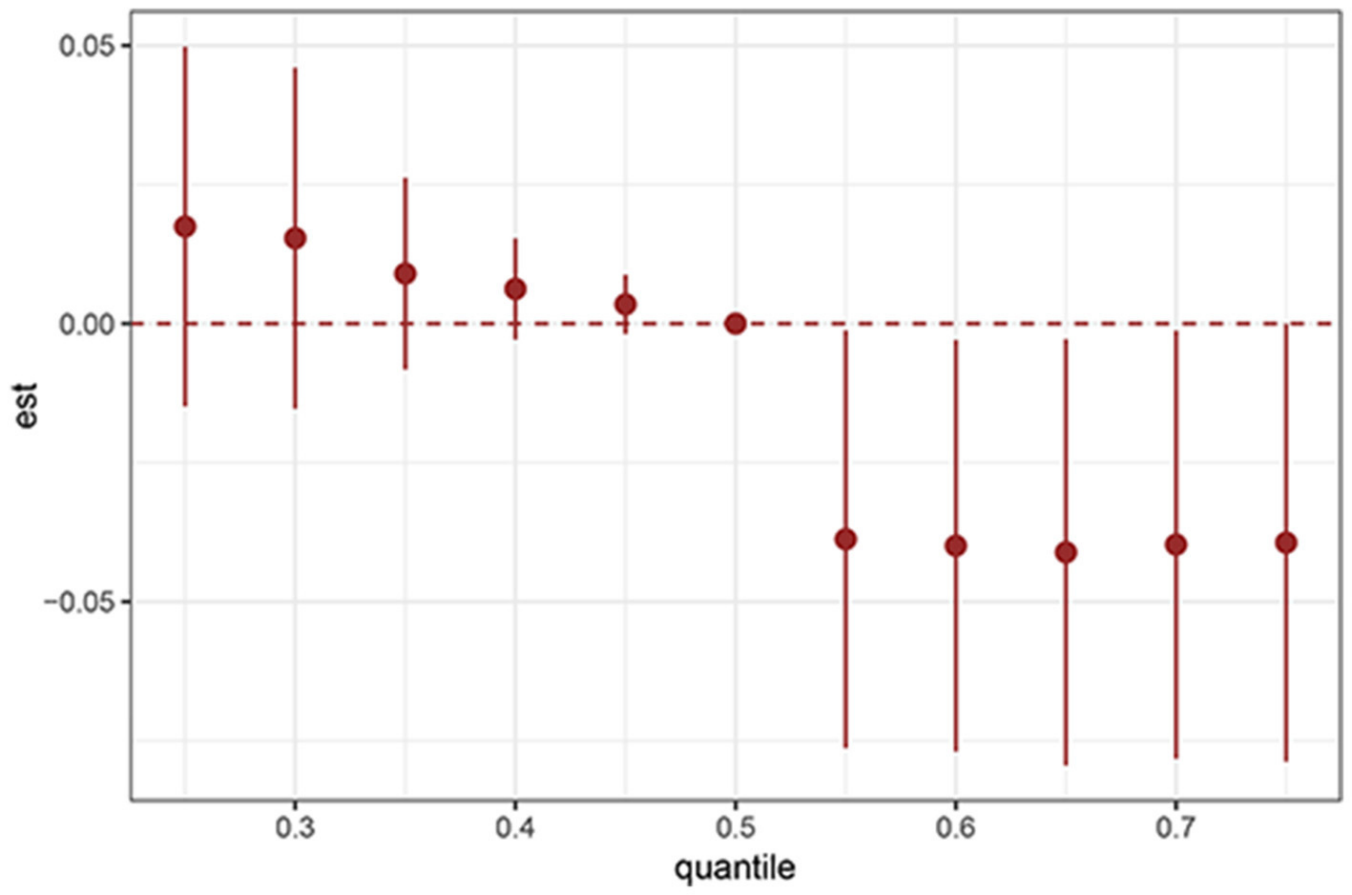
BKMR plots for PFAS and adolescent sleep disorders. Est, estimation.

BKMR analysis revealed a significant negative association between serum PFAS concentrations and the prevalence of sleep disorders among adolescents (Figure 4). The proportion of sleep disorders declined as PFAS exposure increased across quantiles, indicating a cumulative effect of these compounds on sleep health.

WQS regression was used to further assess the combined effects of various PFAS on adolescent sleep disorders. The analysis demonstrated a significant negative association between PFAS compounds and sleep disorders, with distinct effects for positive and negative loadings. The positive effect was not statistically significant (OR: 0.83, 95% CI: 0.6–1.08, *P* = 0.887), whereas the negative effect was significant (95% CI: 0.32–0.85, *P* = 0.009; Table 4).

**Table 4:** Association between PFAS mixture and sleep disorder in adolescents in the WQS model.

**Effect**	**OR (95%CI)**	***P-value***
Positive effect	0.83 (0.06, 10.88)	0.887
Negative effect	0.53 (0.32, 0.85)	0.009

**Figure 5:**
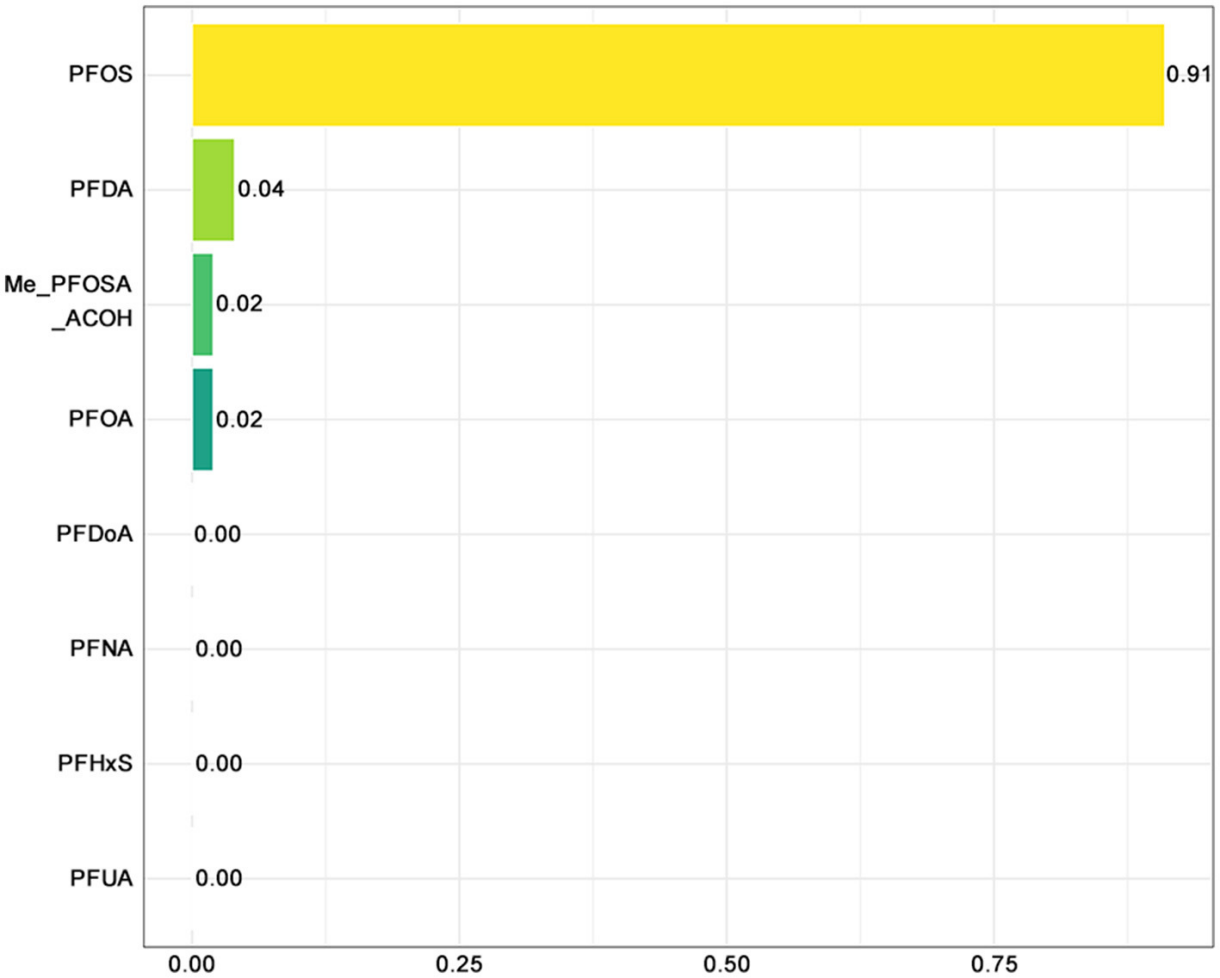
WQS-negative plot of PFAS and sleep disorders. PFOA, perfluorooctanoic acid; PFOS, perfluorooctane sulfonic acid; PFDA, perfluorodecanoic acid; PFHxS, perfluorohexane sulfonic acid; PFNA, perfluorononanoic acid; PFUA, perfluoroundecanoate; PFDoA, perfluorododecanoate; Me-PFOSA-AcOH, 2-N-methyl-perfluorooctane sulfonamidoacetic acid.

Among the PFAS components, PFOS contributed most to the negative association (weight: 0.91), followed by PFOA (0.04) and PFNA (0.02), suggesting that PFOS was the primary effector (Figure 5). In contrast, PFUA, PFHxS, PFDoA, Me-PFOSA-AcOH, and PFDA had negligible weights, implying limited or no contribution to adolescent sleep disorders.

### 3.4 Subgroup analysis

**Figure 6:**
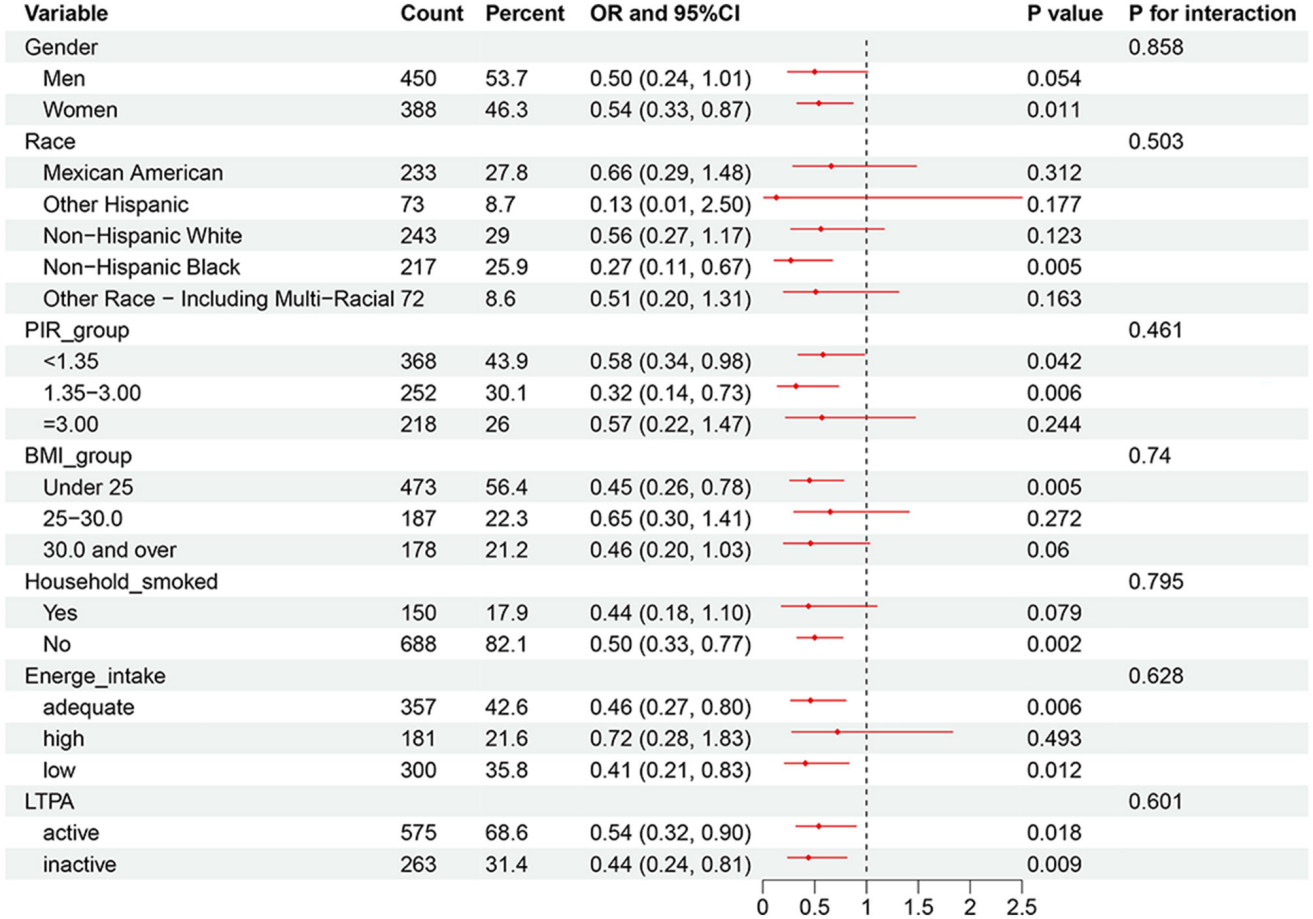
PFOS and adolescent sleep disorders: forest plot for subgroup analyses. OR, odds ratio; CI, confidence interval; BMI, body mass index (kg/m2); PIR, ratio of family income to poverty; LTPA, leisure-time physical activity.

Figure 6 illustrates the association between PFOS exposure and sleep disorders, stratified by sex, race, socioeconomic status, and household smoking status. The results suggest that the protective effect of PFOS may be stronger in males (OR = 0.50, 95% CI: 0.24–1.01) than in females (OR = 0.54, 95% CI: 0.33–0.87). Among racial subgroups, Non-Hispanic White adolescents exhibited a significantly reduced risk of sleep disorders (OR = 0.58, 95% CI: 0.34–0.98). In contrast, individuals from other racial groups, including Multi-Racial adolescents, demonstrated a stronger negative association (OR: 0.32, 95% CI: 0.14–0.73). Moreover, adolescents with PIR values between 1.35 and 3.00 showed a significant reduction in the risk of sleep disorders (OR = 0.32, 95% CI: 0.14–0.73). Finally, adolescents from non-smoking households (OR: 0.46, 95% CI: 0.27–0.80) had a stronger protective effect compared with those from smoking households (OR: 0.50, 95% CI: 0.33–0.77).

## 4 Discussion

To the best of our knowledge, this study represents one of the few investigations into the relationship between multiple PFAS compounds and sleep disorders and is the foremost cross-sectional study to specifically examine this association in adolescents. We conducted a comprehensive analysis using data from the NHANES database, which includes a representative sample of the U.S. population from 2005–2018, to explore potential associations between PFAS exposure and sleep disorders in this age group.

Our findings indicated significant correlations between three types of PFAS (PFOA, PFOS, and PFNA) and sleep disorders in adolescents. Notably, higher serum concentrations of these PFAS were associated with a reduced risk of sleep disorders. This inverse relationship was consistent across various models, even after adjusting for potential confounders such as age, sex, race, socioeconomic status, and lifestyle factors. Additionally, the concentrations of these PFAS compounds were independent of each other, which may be attributed to their distinct sources, environmental persistence, and varying half-lives in the human body (19–21). Moreover, further analysis of PFAS mixtures revealed that cumulative exposure to PFAS compounds showed a protective association against sleep disorders. Among the analyzed PFAS, PFOS emerged as the predominant contributor, accounting for ~91% of the overall effect. This highlights the potential central role of PFOS in shaping the observed associations between PFAS exposure and adolescent sleep health.

PFAS, which are widely used in aerospace, mining, biotechnology, petroleum industry, chemical industry, textiles, and fire extinguishers, have been recognized as endocrine-disrupting chemicals (22, 23). Humans are exposed to PFAS through ingestion, inhalation, and dermal contact (24). A prospective cohort study investigating prenatal exposure to PFAS and infant sleep disorders revealed that prenatal exposure to these substances can adversely impact infant sleep, with effects persisting up to 12 months post-partum (25). The phenomenon is attributed to the ability of PFAS to cross the placental and blood-brain barriers, accumulating in the brainstem, a critical region that regulates essential physiological functions, including sleep (26). In contrast, a study examining the relationship between PFAS and sleep health among adults in the U.S. found no convincing evidence linking legacy PFAS exposure to worsened self-reported sleep health; nevertheless, certain negative correlations between PFAS exposure and sleep disorders were observed (12). These findings align with the results of our study on adolescents, highlighting similarities in the possible associations between PFAS exposure and sleep health across adults and adolescents.

Notably, limited studies have specifically addressed the relationship between PFNA exposures and sleep health in infants. This gap is likely attributed to the challenges associated with collecting serum samples from infants, which is uncommon and often avoided (27). Consequently, knowledge about PFAS exposure and its effects in this age group remains limited. Based on these findings, we hypothesized that the impact of PFAS exposure on sleep disorders may vary depending on exposure timing and age.

An *in vitro* study showed that PFAS may elevate glutamate concentrations, resulting in excitotoxicity, a condition where excessive glutamate activity causes neuronal cell injury and even death (28). Evidence suggests that damage to or death of nerve cells can contribute to sleep disorder development (29, 30). Additionally, PFAS exposure has been linked to increased oxidative stress (31), whereas melatonin, a hormone with antioxidant properties, has been identified as an effective countermeasure (32). When oxidative stress levels rise, melatonin secretion increases to counteract these effects; moreover, melatonin's role in promoting sleep has been well-documented, potentially reducing sleep disorder risk (33).

Based on these findings, we hypothesize that PFAS may influence on sleep through multiple biological pathways. However, further research is needed to explore the precise association between PFAS exposure and sleep disorders and reveal the underlying biological mechanisms driving this association.

Subgroup analyses revealed that PFAS exposure concentrations had a more pronounced impact on female adolescents. Previous research found that PFAS exposure is associated with lower levels of estradiol (E2) and higher levels of follicle-stimulating hormone (FSH) in the serum of female adolescents (34, 35). Lower E2 levels and elevated FSH levels are known to increase the likelihood of nocturnal awakenings, contributing to sleep disturbances (36).

This study had several major strengths. First, this is the first investigation to investigate the relationship between PFAS exposure concentrations and sleep disorders in adolescents. The study found significant associations between three specific PFAS (PFOA, PFOS, and PFNA) and adolescent sleep disorders. We ensured a robust and diverse sample of adolescents by analyzing seven cycles of the nationally representative NHANES dataset. Second, we employed a range of statistical methods to analyze the data. While the individual techniques are not novel, the innovative aspect of this study lies in the comprehensive application of these methods. We first performed individual-level analyses, advanced to mixture analysis, and incorporated WQS regression. WQS regression effectively quantifies the individual contributions of components within chemical mixtures and represents a relatively new approach in PFAS research. Finally, subgroup analyses were performed to examine variations based on sex, race, BMI, and other key confounding factors. Stratified analyses provided a nuanced understanding of the impact on specific sub-populations. For example, our findings suggested that female adolescents and those in households with smoking family members may be more vulnerable to PFAS exposure. These results highlight the importance of targeted interventions in these groups to achieve significant health benefits.

Nevertheless, this study had some limitations. First, we could not establish a causal relationship between PFAS exposure and sleep disorders owing to the cross-sectional nature of this study. Covariates were carefully selected based on existing evidence and previous studies to account for potential confounders that might affect the relationship between PFAS exposure and adolescent sleep disorders. However, it is important to acknowledge that other unmeasured or residual confounders may still exist. For example, environmental factors such as air pollution or noise exposure, which were not included in our analysis, could also influence sleep quality and may interact with PFAS exposure. Second, our findings suggested potential bias in the data related to sleep disorders, particularly regarding information provided by healthcare professionals or self-reported by adolescents. Self-reported data has an inherent risk of inaccuracies, including misreporting or intentional falsehoods. Third, this study was conducted on a relatively small sample of American adolescents, limiting the generalizability of the results to other populations. Whether similar associations would be observed in adolescents from other countries or cultural backgrounds remains uncertain. Future investigations could leverage multinational datasets to expand the adolescent cohort, thereby strengthening the validation of the findings across diverse populations and bolstering statistical robustness, while also considering incorporating a broader range of environmental and lifestyle factors to more comprehensively assess the relationship between PFAS exposure and sleep health. Additionally, corroborative cellular and animal model studies could be employed to further substantiate the observed associations. Such experimental approaches, particularly those focusing on the mechanisms by which PFAS exposure may influence sleep, could provide deeper insights into the biological pathways involved.

## 5 Conclusion

Our findings suggest that exposure to PFAS mixtures may be associated with a reduced risk of sleep disorders in adolescents, with PFOS emerging as the dominant effect component. This association appears to be more pronounced in female adolescents than in males. These results highlight the potential public health impact of PFAS exposure and underscore the urgent need to address this environmental pollutant in the context of adolescent health. Given the widespread presence of PFAS in consumer products and the environment, our study emphasizes the importance of regulatory measures to reduce PFAS exposure, particularly among vulnerable populations such as adolescents. Public health interventions aimed at mitigating PFAS exposure, such as improving water treatment processes and promoting the use of PFAS-free products, could yield significant health benefits.

## Data Availability

The raw data supporting the conclusions of this article will be made available by the authors, without undue reservation.
